# Factors Affecting Thoroughbred Online Auction Prices in Non/Post-Racing Careers

**DOI:** 10.3390/ani13081329

**Published:** 2023-04-13

**Authors:** Madalynn Camp, Michelle L. Kibler, Jennie L. Z. Ivey, Jada M. Thompson

**Affiliations:** 1 Department of Agriculture, Illinois State University, Normal, IL 61761, USA; 2 Department of Animal Science, University of Tennessee, Knoxville, TN 37996, USA; 3 Agricultural Economics and Agribusiness, University of Arkansas, Fayetteville, AR 72701, USA

**Keywords:** thoroughbred, off-the-track thoroughbred, horse racing, willingness-to-pay, equine welfare, hedonic

## Abstract

**Simple Summary:**

Racehorse welfare has generated public attention for both on-track treatment and practices, as well as the fate of racehorses upon completion of their racing careers. As such, the successful transition from racing into sport horse and other disciplines is paramount to the welfare of retired racehorses whose racing career lasts, on average, just 4.5 years. Thoroughbreds are frequently used in sport horse disciplines, and the consistent supply of retiring racehorses provide an affordable and athletic breed option for equestrians. This study analyzes demand for thoroughbreds offered for sale through online, sporthorse auctions. The results show buyer preferences for coat color, age, sex, and those registered with USEF, USHJA, or USEA. These findings support the value of thoroughbreds to buyers interested in sport horse disciplines. The results from this study may help non-profit, off-the-track thoroughbred rehoming facilities, reduce the number of unwanted thoroughbreds, and improve the public image of welfare in the racing industry.

**Abstract:**

Racehorse welfare is a prominent, public issue which affects nearly every aspect of the racing industry. Thoroughbred care after race career completion has garnered increasing attention from the equine industry, general public, and animal welfare groups alike. As the average racehorse’s career lasts just 4.5 years, owner demand for thoroughbreds is essential for post-race careers and acceptable welfare standards. This study utilized data from and hedonic pricing models to analyze buyer demand for thoroughbreds sold in online auctions held from 2012 to 2020. The results indicate buyer preferences for age (*p* < 0.01), sex (*p* < 0.05), and organization registration (*p* < 0.05), with bid price premiums for age and registration status (USEF, USEA, USHJA, etc.) and price discounts for mares compared to geldings and horses listed for non-competition careers (trail, *p* < 0.01). The results of this study confirm and quantify the value potential buyers place on thoroughbreds offered for sale in sport disciplines. The findings may help non-profit organizations charged with rehoming off the track thoroughbreds and reduce the number of unwanted thoroughbreds by illustrating the desired traits and skills within the equine market, thus improving welfare optics overall.

## 1. Introduction

Horse racing is prevalent within the United States, and given the breed restrictions within racing varieties, many purebred thoroughbreds are initially destined for a racing career. In 2018, the American Horse Council estimated that of the 1.2 million horses involved within the racing sector of the United States Equine Industry, over 500,000 of them were thoroughbreds [1]. Furthermore, between 2016 and 2021 The Jockey Club registered an average of 21,000 thoroughbred foals each year and reported nearly 50,000 actively racing thoroughbreds within the United States [2]. A thoroughbred racehorse’s career averages approximately 4.5 years [3], while the average lifespan of a horse is between 20 and 30 years [4]. The actual number of thoroughbreds retiring from the racetrack not selected for racing breeding programs is not known. The Equine Welfare Data Collective aggregates data from self-reported rescue, rehabilitation, and rehoming agencies, but has not yet released reports by breed [5]. For perspective, CANTER has facilitated the rehoming of over 25,000 retired thoroughbreds through either CANTER owned horses or by facilitating trainer/owner horse sale listings [6]. Current industry circumstances, including the restriction of the USDA inspection of equine processing facilities, high numbers of unwanted horses, and scrutiny of equine welfare from the general public and industry members alike [7] have increased the need for marketable skills and options for thoroughbreds that are not or are no longer utilized for racing. Furthermore, the restriction of USDA inspection of equine processing facilities and the prevalence of thoroughbreds entering the slaughter pipeline can complicate welfare implications for horses in post racing careers, despite efforts from tracks and racing jurisdictions to prevent horses from being sold for processing purposes [7,8,9,10].

Attention on the post-racing welfare of thoroughbreds has increased and created various outlets for placement. The scrutiny of horse racing itself poses unique challenges for second career placement. In a poll assessing welfare, injury incidence, fatalities, and other pressing issues affecting the racing industry, 31% of United States residents likely to vote in upcoming elections felt that racehorses are not treated in humane ways [11]. Securing care for post-racing Thoroughbreds can be challenging due to the number of Thoroughbreds leaving active racing each year, available adoption/rehoming programs, and industry demand for purchasing horses for alternative careers. Furthermore, horses that are not marketable within available outlets may face poor welfare situations, which complicates the tenuous nature of the perception of horse racing [12]. The Jockey Club encourages the adoption of retired racehorses who are not selected to enter breeding programs and endorses their suitability for second careers (i.e., sport disciplines, barrel racing). Various placement or rehoming programs have incorporated training, certifications, or incentives for individuals and organizations who work with off-the-track thoroughbreds (OTTB), such as The Thoroughbred Aftercare Alliance, Thoroughbred Charities of America, and the Thoroughbred Incentive Program [13,14,15]. For example, the Thoroughbred Incentive Program offers thoroughbred only competitions to encourage equestrians to purchase and retrain retired racehorses, even hosting championships in disciplines such as hunter, jumper, dressage, and western. The addition of marketable skills through alternative discipline training is useful for increasing desirability; however, the value of additive skills is largely unknown.

While existing research has investigated the demand for retired/OTTB offered through adoption facilities [16], there is limited information on price determinants for thoroughbreds sold outside of race-specific auctions (such as Fasiq-Tipton and Keeneland) and sales [17,18,19,20]. Understanding market demands can aid in identifying the desired characteristics for thoroughbreds entering alternative disciplines, which may in turn improve welfare for this breed sector by better aligning buyer demand with available market supply [7]. Thus, the objective of this study was to assess buyer demand and price determinants for thoroughbreds offered for sale through non-racing online auctions.

## 2. Materials and Methods

### 2.1. Data Collection

Data on thoroughbreds was collected from 39 online auctions offered through an online auction service [21]. Auctions were held between 2012 and 2020, and data were accessed between April and December 2020 through publicly available archives. All auctions within this date range were examined for the listing and successful sale of thoroughbred horses, with the exception of breed specific sales that would preclude the entry of thoroughbreds (such as the Chincoteague pony or Rancho Corazón auctions). The thoroughbreds represented in these auctions comprised all age ranges, including both thoroughbreds transitioning into second careers and those who have already transitioned. It was not possible to determine which category each horse represented and when each horse retired from the racetrack based on the reported sale information. Data were collected to create a dataset on equid and auction characteristics including age, sex (gelding or mare, no stallions listed), colors recognized by the Jockey Club (bay/brown/black, chestnut and bray/roan), height, binaries for pre-sale veterinary information, and for any health concerns including major surgery, past lameness, other defects/abnormalities/blemishes, and cribber/weaver. Insufficient observations for dressage level and jumping height suffered from low reporting, and as a result were not included in the model. Information on equid training was collected and included professional memberships with the Jockey Club, the United States Equestrian Federation (USEF), the United States Hunter Jumper Association (USHJA), and the United States Eventing Association (USEA) (Jockey Club registration and USEF/USHJA/USEA membership) as well as disciplinary training (Dressage, Jumper, Hunter Under Saddle, Trail, and English General Riding). In some instances, more than one membership or disciplinary training classification was listed, and thus was quantified accordingly. Insufficient observations for dressage level and jumping height suffered from low reporting, and as a result were not included in the model. Memberships provide information on second career skills the horse may already have been introduced to or trained for, and could augment specifically listed disciplines in the sale advertisement.

In addition to horse characteristics, information presented on the sale advertisement is an important component for sellers in online auctions [22]. Online auctions may not provide the buyers the opportunity to view horses in person, so sellers attempt to overcome these limitations by providing images, video, and detailed information regarding the horse. Auction images were classified as Non-Action Shot, stationary photographs of showing the equid’s conformation through front, back, side, and hoof pictures, or Action Shot, depicting the horse moving or working. Quantities of each image classification were obtained. Additionally, video content within the sale advertisement was assessed, and when a video(s) was present, the quantity of videos was recorded. Finally, advertisements containing descriptions or notes were quantified by counting the number of lines of notes, which could include information such as racing history, show experience, athletic capabilities, or demeanor. Screen resolution was kept consistent among data collection to ensure the consistent quantification of notes between auctions and displays.

Listings were accessed and available data was recorded for initial inclusion screening. Specifically, for a listing to be included in study analyses, it must have met the following criteria: (1) The breed was listed as thoroughbred only, with no crossed or mixed breeds, (2) a successful sale was indicated and a final sale price was listed in the advertisement, and (3) the horses sold were located within the United States. No restrictions were placed on age or sex for study inclusion.

### 2.2. Statistical Analysis

All statistical analysis including summary statistics and model results were calculated using Stata 17 software [23]. Specifically, summary statistics included calculations of means, minimums, maximums, and standard deviations for each variable. To understand the market for sport discipline thoroughbreds and provide the market with realistic value expectations to help the transition to a second career, a hedonic pricing model was used in accordance with previously published methods [22,24,25,26,27], where models are used for decomposing prices into individual attributes to help future market participants with regard to better price expectations. The empirical model used in this analysis is described in equation 1 below as:

(Bid)= α + βHorse Characteristics + γ Auction Characteristics + ε,(1)

where the bid price is regressed on a constant term (α), a series of horse characteristics and auction characteristics with vectors of respective coefficients (β and γ). Horse and auction characteristics are described in the data section and in Table 1. The independent error term is represented by ε. The models were tested for homoscedasticity and then remodeled with robust standard errors to address heteroskedasticity [28]. Significance was detected when *p* < 0.05, and trends were identified when *p* < 0.1. Where appropriate, data are reported as the mean ± SD unless otherwise stated. To avoid multicollinearity for binary variables in the model, Gelding, bay/black/brown, and English General Riding were excluded from the model. The excluded variables serve to provide base comparisons for sex, coat color, and discipline-independent binary variables.

## 3. Results

Of the 245 thoroughbred breeds listed for sale between 2012 and 2020, 57 did not have a successful sale bid price, 11 did not have a height listed, 5 were from Canada or had an unlisted location, and 2 were dropped because they were limited sample outliers. Thus, a total of 170 sale advertisements for thoroughbred horses met the inclusion criteria and comprised the study population.

The thoroughbreds sold varied in physical characteristics and sale listing information (Table 1). Horses (n = 170) ranged in age (8.64 ± 3.68 years, min = 1 year, max = 20 years, n = 170) and were mostly mares (107; 63%). Bay/brown/black was the predominant color identified (n = 105, 62%), followed by chestnut (n = 44, 26%). Thoroughbreds in this study stood 164.48 cm ± 5.47 tall (min =147.32, max = 180.34). All horses offered for sale (n = 170) had at least one picture presented on the sale ad. Only 16% (n = 27) of the horses provided pre-sale veterinary information. In terms of second careers or experience potential, Jumper (n = 63) was most commonly reported, followed by Hunter Under Saddle (n = 54) and Trail (n = 43). The hunter jumping height was reported on 30 listings, where jump height ranged from 68 to 106.68 cm, whereas jumper height was reported on 17 listings and spanned from 70 cm to 142.2 cm. The dressage level was only reported in six advertisements, ranging from training, first, and second levels. Due to the low sample size within each jumping height or dressage level, these variables were not included in the hedonic model. Horse registration was indicated through The Jockey Club (n = 59) and USEF/USHJA/USEA (n = 17) membership. For this analysis, pictures were categorized into action pictures and non-action pictures, where at least one horse had no posted non-action or no posted action picture. Non-action pictures were used in 120 listings displaying 1.96 ± 1.71 pictures (min = 0 picture, max = 11 pictures). Action pictures were used in 140 listings which contained 0.82 ± 0.38 pictures (min = 0 pictures, max = 6 pictures). Videos were included in 154 listings, with some having as many as four videos. All sale ads included notes which ranged widely between a minimum of three to a maximum of 72 lines (16.62 ± 11.63 lines).

### Hedonic Model Results

The hedonic model results are presented in Table 2. The age of the horse when auctioned was statistically significant (*p* < 0.01) and had a positive effect on bid price (USD 430.50); however, the squared age term indicates that this increase is at a decreasing rate (USD −22.94, *p* < 0.01). Holding other horse characteristics constant, a horse’s sale price increases until price peaks at age 9 and then begins to decrease thereafter. Gender influenced bid price (*p* < 0.05), where mares were found to be valued an average of USD 924.08 less than geldings. A trend for chestnut and gray or roan horses to be valued over USD 1000 (*p* < 0.10) more than bay, brown, or black horses was detected.

Registration memberships to The Jockey Club and USEF/USHJA/USEA help inform owners of the horse’s current or past experience. Membership to the USEF, USHJA, or USEA contributed to Thoroughbred value, adding USD 1967.33 ± USD 820.24 to the bid price (*p* < 0.05).

The type of disciplines the horses were either trained in or were prospects for included dressage, jumper, hunter under saddle, trail, and English general riding. When compared to English general riding, trail was the only variable found to significantly (*p* < 0.01) influence final sale price, where horses with trail listed were valued USD 1599.46 less than English general riding horses on average.

## 4. Discussion

Given the scrutiny surrounding Thoroughbred racing and the Social License to Operate, any improvement on horse welfare, marketable skill increase, and long term placement of thoroughbred horses may help improve the overall perception of the racing industry sector [7]. While not all thoroughbreds within the United States have actively raced, much of the population may have been involved with the industry through initial breeding or production [1,2]. Even without a racing background, the thoroughbred breed is so closely linked to racing that negative welfare or publicity could be associated with the racing sector. Attention to aftercare of OTTBs through sales, rescue, retraining, and or retirement has increased in recent years, where factors relating to length of stay in rescue/retraining facilities and long term satisfaction with thoroughbred ownership have been evaluated [7,16,29]. This study assessed buyer demand and price determinants for thoroughbreds offered for sale through non-racing online auctions. A further understanding of the factors relating to owner demand, skills, and overall desire for thoroughbreds within the equine market plays an important role in understanding how horses can be marketed and sold or successfully placed and maintained long-term within new careers.

Hedonic pricing models have been used in the literature extensively and specifically for equine market analyses. For example, within the thoroughbred industry, Hansen and Stowe [24] determined the impact of thoroughbred weanling characteristics on price when sold at auction, Neibergs [25] investigated the demand characteristics of broodmares, Stoeppel and Maynard [26] looked specifically at thoroughbred broodmares in foal and how their characteristics affected their auction price, and Stowe and Kibler [16] investigated the characteristics of thoroughbred racehorses adopted from nonprofit organizations. Studies pertaining to non-thoroughbreds at auction include Kibler and Thompson’s [22] analysis of the price determinates for stock-type horses and Taylor et al.’s [27] calculation of the price determinants of show quality quarter horses.

While numerous factors can be considered important to individual buyers, this study determined age, sex, color, registration, and discipline to be significant factors in thoroughbreds’ online auction price. Buyers place increasing value on a horse as it ages though at a decreasing rate, indicating that as the horse ages, there will be a turning point, or an age, where value begins to decrease as the remaining years a horse can be used for competition becomes shorter [27]. Parameter estimates indicate this turning point to be at age 9, and horse value declines each additional year afterward. Price decline with age has been documented in other breeds, and displayed a similar peak in mare and gelding prices for quarter horses sold in quality auctions and horses sold at a Minnesota auction [27,30]. Based on hedonic modeling results by age 19, horse value is negative where, with other horse characteristics held constant, a horse would have to be given away. However, in many cases, a horse’s value will continue to be greater than zero, as other characteristics of the horse have value to horse owners (training, experience, etc.). This places the average racehorse retirement age at approximately 4.5 years, the halfway point to the optimal value based on age to potential buyers and, when time is spent retraining these horses for second careers, the horse is still considered to be at prime age. Findings from previous studies show the time until adoption of off-the-track thoroughbreds indicates that older horses spend more time at adoption facilities than younger horses, highlighting the importance of thoroughbreds retiring and transitioning to second careers at a younger age in order to attract potential buyers [16].

The buyers showed a preference for geldings over mares, where mares were valued USD 924.08 less than geldings. This result contradicts findings from stock and quarter horse studies, where buyers placed premiums on mares compared to stallions and geldings [22,27], perhaps indicating a heterogeneity among segments of the equine sales market. There was a larger number of mares in this study, which is a function of the sex of the horses that sellers were offering for sale in this format over the study period. Coat colors of chestnut and gray/roan were preferred (USD 1071.07 and USD 1054.05, respectively) to bay/brown horses. Interestingly, gray thoroughbreds placed for adoption off-the-track were found to spend less time at an adoption facility, but were more likely to be returned to the adoption facility though the authors did not determine a cause for this occurrence [16]. This result is not surprising, as chestnut and gray or roan horses are not as common as bay, brown, or black horses. In this study, approximately 26% of the horses were chestnut and 12% of the horses were gray or roan, making up less than half of the total sample. Existing studies have found similar results in buyer preference for less common coat colors and/or patterns for stock type horses and wild mustangs [22,31], though color preferences for stock type horses contributed to price discounts for gray and sorrel horses [22], perhaps supporting a preference difference between disciplines.

Horses registered with national breed or discipline registration are preferred by buyers [22]. While registration for The Jockey Club did not affect the bid price, registration within USEF, USHJA, or USEA was found to bring in a premium bid. This indicates that buyers place a premium on thoroughbreds with showing or competition experience, and registration with these organizations affords buyers the opportunity to look up past competition experience and placings. The lack of significance for thoroughbreds with Jockey Club registration indicates neither a preference for or against Thoroughbreds who were bred for racing, which may illustrate that buyers viewed all thoroughbreds similarly despite prior racing history or association with The Jockey Club.

Buyers have shown a variance in how they value horses suitable for different disciplines [22,27]. In an assessment of sport horse sale outcomes, professional training did not increase the likelihood of a successful sale, but did increase the commanded price [32]. Furthermore, in the same study, horses with less experience reported in the sale advertisement were more likely to sell compared to horses with more experience reported [32]. Results from the current analysis indicate that thoroughbred horses listed with experience or potential in trail were found to be valued less than horses listed as English general riding, suggesting that auction site bidders in these online auctions could be searching for a more competitive horse, or that trail riding was viewed as a discipline that required less formalized training than other types. Thoroughbred horses with trail riding listed in sale advertisements also brought lower sale prices compared to other disciplines for use in adult recreational riding markets [33]. Additionally, lower market values were observed for stock horses sold in online auctions, where on average trail horses yielded nearly USD 1600 less than horses with other disciplines listed [22]. This connection is of particular merit, as thoroughbreds retiring from the racetrack often retire with an existing injury [34]. Injuries limiting the discipline or upper-level suitability for a particular discipline (eventing or jumping, for example) have shown to be less desirable to potential adopters, as these horses experience longer stays at adoption facilities [16]. As the auction site is named ‘SportHorseAuctions.com’, it is possible that buyers using this site sought horses with specific sport horse training, and this may have also influenced the bid price for horses listed with trail experience.

Competitive dressage, hunter jumper, and jumper disciplines inherently contain distinctive levels or jumping requirements within shows and other event types. Unfortunately, within this study, insufficient observations prevented jumping height and dressage level from being analyzed for independent effects on price premiums; however, it is possible that these variables may influence overall price. The continuous nature of the jumping and dressage levels coupled with the limited sample with the information listed provide too few observations for an analytical estimation of its hedonic value. While we captured the effects of binarily reporting dressage, which was insignificant, future work could try to collect more complete disciplinary performance metrics to better understand their value. In other breeds, the number of championships or top placing at the American Quarter Horse Association World Show and number of futurity championships or placings yielded a higher sale price [27]. Similarly, for each additional point earned within registered shows during the same year as the sale increased the price premium by 2% [27]. Logically, as a horse’s ability to compete in dressage levels and jumping height progresses, the amount of training would consequently increase. Similarly, given the increased nature of horses in post-racing careers to develop performance limiting conditions, it may be of value to assess the performance level across competitive disciplines to determine how the competitive level affects premiums (eventing level, dressage level, jumping height, etc.) [22,29].

The inclusion of photos, videos and lines of notes or descriptions were not found to be influential in the sale price; however, previous studies found lines of notes, back pictures, side pictures, action shots, and videos to be significant [22]. Of the equids listed for sale in the study, every sale ad included at least one photo, and the mean number of note lines was 16.62. Furthermore, the size of the advertisement with specifications to eighth or quarter page ads was an influencing factor to increase the sale price of horses sold for recreational disciplines [33]. It is possible that the results in this study could imply that videos, pictures, and descriptions are standard types of advertising information within online auctions, or play a larger role within different targeted disciplines within this venue.

Pre-sale veterinary information could include health information along with the horse’s soundness, and can inform the buyer of any relevant health concerns [34]. When a seller discloses this type of information, the buyer can make a more informed decision pertaining to the horse’s health and the potential financial responsibility and management needs for that specific animal. While pre-sale veterinary information was insignificant in the current study, previously completed work has illustrated the differential impacts of health-related disclosures on sale prices. In thoroughbred horses with previous hospitalization, no differences were detected in racing sale price from foal through 2 years of age [35]. Conversely, the disclosed health information for thoroughbred yearlings influenced the price for horses that sold at lower prices [36]. Furthermore, individuals that adopted OTTBs reported increased musculoskeletal injuries and gastrointestinal, behavioral, and foot/hoof issues than non-OTTBs [29]. Within the reported sale advertisements assessed in the current study, a minimal amount of horses were listed with health concerns (16%, n = 27). The sellers may have elected not to disclose health information due to apprehension of potential sale impact; however, health disclosure is an important factor for buyers to fully understand the anticipated financial burden for known health problems.

Given small sample size, individual health concerns (cribber, weaver, past surgeries, etc.) listed for each horse were categorized as one binary variable, thus, it is possible that buyers have preferences for avoiding specific health concerns or value horses with these attributes less, but individual impacts could not be investigated. In an assessment of online auction sale outcomes, negative horse traits (stereotypic behaviors, cribbing, weaving, stall walking, etc.) did not influence buyer demand for stock type horses [22]. Similarly, the incidence of stall walking or weaving did not influence the performance in active racing Thoroughbred horses [37]. While stereotypic behaviors are often regarded as undesirable traits within the equine industry and frequently result from poor welfare situations, additional work to investigate the connection between thoroughbred welfare, stereotypic behaviors, and non- or post-racing careers is warranted [37,38].

## 5. Conclusions

The successful transition of retired thoroughbred racehorses is paramount to the image and social license to operate for the thoroughbred racing industry. While successful transitions may be measured in various ways (e.g., the number of retired racehorses placed in non-racing homes), buyer demand for thoroughbreds who have already begun second careers may indicate the long-term successful placements of OTTBs and non-racing thoroughbreds. This study found that thoroughbred buyers have preferences for coat color, age, sex, riding discipline, and discipline registry status (USEF, etc.). Future works should consider the demand for thoroughbreds in second careers within the broader context of all breeds and disciplines (e.g., sport horses) and the implications of perceived welfare conditions. Further investigations of the length of racing careers and/or racing industry connections, individual health concerns, jumping height, and training or discipline level on buyer demand is warranted. It is not known how prior racing history, including length of racing career, racing industry connection, race earnings, etc., may impact buyer demand for OTTBs, and therefore future work could expand the scope of data collection to all information, not just what is listed on a sale ad.

## Figures and Tables

**Table 1:** Thoroughbred Online Auction Data Description and Summary, 2012–2020 (n = 170).

Variable Name	Variable Description ^1^	Mean	Standard Deviation	Min	Max
Bid Price (USD)	Highest bid the horse received	$2439.12	$2726.45	$100.00	$20,000.00
Auction Year	Year of auction listing	2016.65	2.28	2012	2020
Age	Age of horse when auctioned	8.64	3.68	1	20
Gender					
Gelding	=1 if horse is a gelding	0.37	0.48	0	1
Mare	=1 if horse is a mare	0.63	0.48	0	1
Color					
Bay/Brown/Black	=1 if horse color was listed bay, black, brown	0.62	0.49	0	1
Chestnut	=1 if horse color was listed chestnut	0.26	0.44	0	1
Gray/Roan	=1 if horse color was listed gray or roan	0.12	0.33	0	1
Height (in cm)	Height in centimeters	164.78	5.47	147.32	180.34
Auction Images					
Non-Action Picture	Count of non-action pictures ^2^ of the horse	1.96	1.71	0	11
Action Picture	Count of action pictures of the horse	0.82	0.38	0	6
Video	Count of videos of the horse	1.82	1.14	0	4
Pre-Sale Vet Information	=1 if pre-sale veterinary information was available	0.16	0.37	0	1
Health Concern	=1 if the horse had any health concerns ^3^	0.34	0.47	0	1
Jockey Club Registration	=1 if the horse was advertised as Jockey Club Registered	0.35	0.48	0	1
USEF/USHJA/USEA Membership	=1 if the horse was advertised with a USEF/USHJA/USEA Membership	0.1	0.3	0	1
Notes	Count of lines in note section ^4^	16.62	11.63	3	72
Discipline Types					
Dressage	=1 if the horse participated or was a prospect for dressage	0.25	0.43	0	1
Jumper	=1 if the horse participated or was a prospect for jumper	0.37	0.48	0	1
Hunter Under Saddle	=1 if the horse participated or was a prospect for hunter under saddle	0.32	0.47	0	1
Trail	=1 if the horse participated or was a prospect for trail	0.25	0.44	0	1
English General Riding	=1 if the horse participated or was a prospect for English general riding	0.19	0.4	0	1

^1^ All binary variables were 1 if listed, and zero otherwise; ^2^ Includes if the horse was advertised with a front, back, or side picture; ^3^ Includes Major Surgery, Past Lameness, Other defects/abnormalities/blemishes, and Cribber/Weaver. ^4^ Includes a count of the lines of notes a seller included in the sales ad.

**Table 2:** Hedonic Modeling Regression Results for Thoroughbred’s Sold in Online Auctions 2012–2020.

Variable Name	Coefficient Estimates (USD)	Standard Error
Auction Year	31.53	−107.05
Age	430.50 ***	−158.69
Age * Age	−22.94 ***	−6.74
Mare ^1^	−924.08 **	−427.84
Chestnut ^2^	1071.07 *	−555.28
Gray Roan	1054.05 *	−536.41
Height (cm)	10.59	−28.93
Non-Action Picture	−187.58	−124.83
Action Picture	−17.7	−112.4
Videos	207.86	−177.81
The Jockey Club	−356.36	−425.98
USEF USHJA USEA	1967.33 **	−820.24
Pre-Sale Vet Info Provided	811.19	−779.98
Notes ^3^	14.49	−22.3
Health Concern	−182.39	−537.39
Dressage	300.82	−740.88
Jumper	1105.74	−703.87
Hunter Under Saddle	229.83	−463.89
Trail	−1599.46 ***	−604.58
Constant	−65,151.94	216,253.10
Number of Observations	170	
F(19,150)	2.53	
Prob > F	0.001	
R-squared	0.2548	

* *p* < 0.10, ** *p* < 0.05, *** *p* < 0.01; ^1^ Gelding and stallions are in the baselevel. ^2^ Baselevel is bay/brown horses. ^3^ Count of the lines of notes included in the sale ad by the seller.

## Data Availability

Data for this study were collected from the sales results link through SportHorseAuctions.com (available online at https://sporthorseauctions.com/, accessed on 25 July 2020).
